# Association between examined lymph node count and survival in resectable cervical cancer: a retrospective analysis using SEER data

**DOI:** 10.3389/fonc.2025.1553587

**Published:** 2025-05-29

**Authors:** Jing Gao, Wanchun Yin, Zhiqing Zhang, Qin Zhou

**Affiliations:** ^1^ Department of Gynecology, the First Affiliated Hospital of Chongqing Medical University, Chongqing, China; ^2^ Department of Gynecology, People’s Hospital of Chongqing Liangjiang New Area, Chongqing, China; ^3^ Department of Endocrine Ward, Huadong Hospital Affiliated to Fudan University, Shanghai, China

**Keywords:** cervical cancer, examined lymph node, restrictive cubic spline, cancer-specific survival, SEER

## Abstract

**Background:**

The optimal examined lymph node (ELN) in resectable nonmetastatic cervical cancer patients remains controversial.

**Methods:**

A total of 7435 N0 patients and 1385 N1 patients were enrolled from the Surveillance, Epidemiology, and End Results database. The relationship between ELN and cancer-specific surcical (CSS) was evaluated by restrictive cubic spline (RCS) method. Survival analysis was performed by using Kaplan–Meier method.

**Results:**

The median ELN count decreased over years both in N0 and N1 patients. The RCS illustrated nonlinear relationships between ELN counts and prognosis for N0 patients (nonlinearity, p= 0.026; optimal ELN: 13) and N1 patients (nonlinearity, p= 0.024; optimal ELN: 14). Patients were divided into ELN adequate and limited groups according to the optimal cutoff of ELN. The 5-yr and 10-yr survival rates were 94.4% and 92.5% for N0 adequate patients, and 93.9% and 90.0% for N0 limited patients. The 5-yr and 10-yr survival rates were 73.8% and 70.3% for N1 adequate patients, and 68.6% and 63.5% for N1 limited patients. For N0 patients, no survival benefit was found in additional adjuvant treatment. For N1 adequate patients, those with adjuvant radiotherapy obtained greatest survival benefit. For N1 limited patients, those with adjuvant radiotherapy or radiotherapy plus chemotherapy obtained better survival.

**Conclusions:**

Nonmetastatic cervical cancer patients with clinical N0 and N1 stages who had at least 13 and 14 ELN counts, respectively, showed better long-term survival. Further prospective studies are needed to validate the association between ELN count and long-term survival.

## Introduction

Cervical cancer is a common malignancy that threatens women’s health (1). According to the Global Cancer Statistics 2020 (2), the incidence and mortality of cervical cancer both rank fourth in women. In 2018, the International Federation of Gynecology and Obstetrics (FIGO) classification system emphasized the role of lymph node metastasis (LNM), and incorporated LNM into the staging criteria, defining cervical cancer with LNM evaluated by imaging or pathological examination as stage IIIC, including stage IIIC1, the presence of LNM in the pelvic region, and stage IIIC2, the presence of para-aortic lymph node metastases (3).

Radical hysterectomy and lymphadenectomy are the most common treatments for nonmetastatic cervical cancer (4). Comprehensive harvesting of lymph node helps for evaluating the status of lymph nodes metastasis, and enhances the precision of clinical staging, which contribute to treatment decision-making. However, the results of studies focusing on the exact number of examined lymph node (ELN) were controversial. A few studies demonstrated positive association between a higher ELN count and a better survival (5–7). While there were studies supported that extensive lymphadenectomy was unfavorable to the prognosis through leading to more postoperative complications and damaging the immune system (8). Besides, a study found no prognostic effect of ELN count (9). Hence, the optimal number of ELN for reliably evaluating the pathological N category in cervical cancer patients needs further clinical evidence.

In recent years, several studies have indicated a nonlinear association between ELN count and survival in various malignancies (10–13), by using the restricted cubic spline (RCS) method. In these studies, an optimal ELN count was proposed and that could distinguish patients at high risk of death from those with survival benefits. For patients with cervical cancer, the optimal ELN count has not been well addressed. Hence, in this study, we aimed to explore the optimal ELN count for resectable cervical cancer patients and investigate the prognostic role of the optimal ELN count, by using a large dataset from Surveillance, Epidemiology, and End Results (SEER) dataset.

## Methods

### Patients

**Figure 1:**
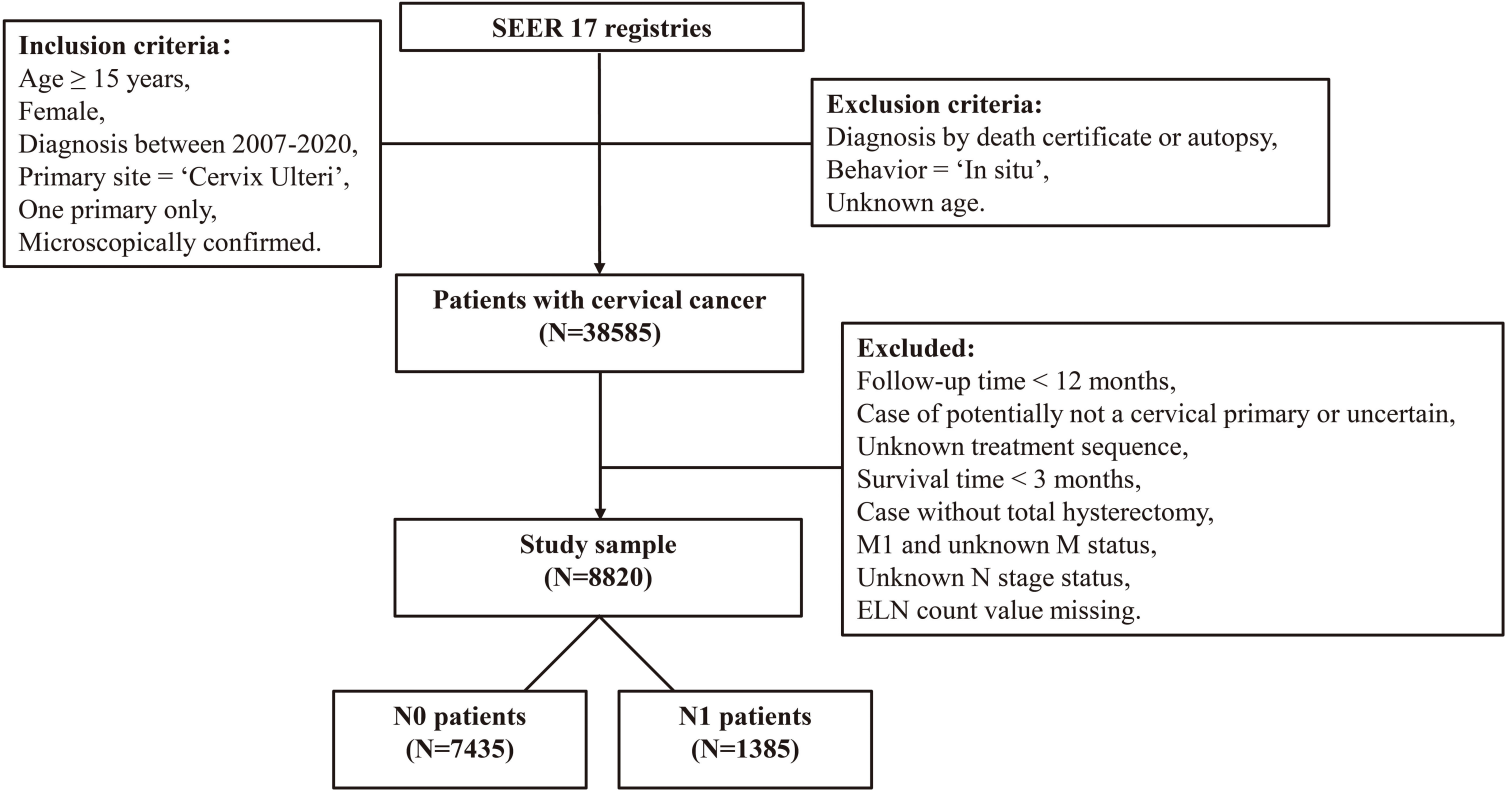
The flowchart of the study design.

In this study, we included resectable non-metastatic cervical cancer diagnosed between 2007 and 2020. The SEER 17 registry was used to screen eligible patients for this study. The inclusion and exclusion criteria were presented in **Figure 1**. In brief, cervical cancer patients who received radical hysterectomy and at least one retrieved lymph node were enrolled. The information on the sequence between chemotherapy and surgery was only available since year 2007, and at least a follow-up of 12 months was guaranteed, the selection of patients was limited to those diagnosed between 2007 and 2020. Then all patients were divided into N0 group and N1 group according to the imaging evidence of node status available in SEER database, where N0 indicated no radiological evidence of LNM and N1 indicated the presence of LNM.

### Study point

The study point of this study was cancer-specific survival (CSS), which was defined using the SEER survival time and SEER cause-specific death classification. The cutoff for the last follow-up was December 31, 2021.

### Study design and statistical analyses

A description analysis of study population, and the differences in clinical characteristics between N0 patients and N1 patients were performed. Categorical variables were presented as numbers and proportions, and continuous variables were presented as median with interquartile range (IQR). The statistical methods included Pearson’s Chi-squared test and Wilcoxon rank sum test. The distribution of ELN in N0 patients and N1 patients were described. The changes of median ELN count over year were calculated.

The RCS method, based on a multivariable Cox model with three knots at the 10th, 50th and 90th percentiles of ELN, was applied to present the nonlinear prognostic profiles between ELN and CSS. This sophisticated method constitutes a validated and robust strategy for identifying critical inflexion points in risk functions, thereby providing insightful understanding into the prognostic profiles of ELN in resectable non-metastatic cervical cancer patients (14).

According to the optimal cutoff of ELN, patients were divided into ELN adequate group and ELN limited group. Patients whose ELN counts were equal to or above this threshold were classified as the ELN adequate group, while those with ELN counts below the threshold were classified as the ELN limited group. This stratification aimed to distinguish patients who potentially benefit from sufficient lymph node evaluation from those who may not. Survival analyses were performed between adequate group and limited group both in N0 patients and N1 patients. The Kaplan–Meier method and the log-rank test were used to compare the survival difference. The hazard ratio (HR) and 95% confidence interval (CI) was calculated by Cox model. Additionally, the survival differences among different adjuvant treatments in different ELN status subgroups were performed.

All data were extracted from SEER*stat software (version 8.4.3; https://seer.cancer.gov/seerstat/). All statistical analyses were performed through R software (version 4.4.0; https://www.r-project.org). A two-sided p-value <0.05 was considered to indicate statistical.

## Results

### Patient characteristics

From 2007 to 2020, a total of 8820 eligible patients were enrolled, including 7435 N0 patients and 1385 N1 patients. Compared to N1 patients, N0 patients had a higher proportion of younger age (15–45 years, 54% vs. 50%, p=0.016), married status (52% vs. 47%, p=0.002), histology of adenocarcinoma (41% vs. 23%, p<0.001), Grade I (18% vs. 5%, p<0.001), smaller tumor size (≤ 20 mm, 46% vs. 16%, p<0.001), but lower proportion of histology of squamous cell carcinoma (SCC) (52% vs. 65%), Grade III (24% vs. 40%), greater tumor size (21–40 mm, 24% vs. 40%; > 40 mm, 12% vs. 34%). Approximately 3% of N0 patients and 7% of N1 patients (p<0.001) received neoadjuvant treatment. Most of N0 patients (76%) received no adjuvant treatment after surgery. For N1 patients, 14% of patients received no adjuvant treatment. But 9%, 5%, and 72% received radiotherapy, chemotherapy, radiotherapy and chemotherapy, respectively. The details of clinical characteristics were presented in **Table 1**.

**Table 1:** Patient characteristics.

Characteristics	Overall, N = 8820	N0 patients, N = 7435	N1 patients, N = 1385	p-value*^3^*
Year of diagnosis				0.084
2007-2009	2302 (26)	1924 (26)	378 (27)	
2010-2015	4391 (50)	3686 (50)	705 (51)	
2016-2020	2127 (24)	1825 (24)	302 (22)	
Age, years				0.016
15-45	4666 (53)	3975 (54)	691 (50)	
≥45	4154 (47)	3460 (46)	694 (50)	
Race				0.098
White	6938 (79)	5850 (79)	1088 (79)	
Black	680 (8)	589 (8)	91 (6)	
Other	1202 (13)	996 (13)	206 (15)	
Marital status				0.002
Married	4540 (51)	3887 (52)	653 (47)	
Ever	1466 (17)	1209 (16)	257 (19)	
Single	2386 (27)	1974 (27)	412 (30)	
Other	428 (5)	365 (5)	63 (4)	
Histology type				<0.001
ADC	3365 (38)	3045 (41)	320 (23)	
SCC	4764 (54)	3871 (52)	893 (65)	
ASC	460 (5)	349 (5)	111 (8)	
Other	231 (3)	170 (2)	61 (4)	
Tumor grade				<0.001
Grade I	1373 (16)	1300 (18)	73 (5)	
Grade II	3243 (37)	2708 (36)	535 (39)	
Grade III	2337 (26)	1774 (24)	563 (40)	
Grade IV	129 (1)	91 (1)	38 (3)	
Unknown	1738 (20)	1562 (21)	176 (13)	
Tumor size, mm				<0.001
≤ 20	3606 (41)	3384 (46)	222 (16)	
21-40	2407 (27)	1849 (24)	558 (40)	
> 40	1363 (16)	890 (12)	473 (34)	
Unknown	1444 (16)	1312 (18)	132 (10)	
Neoadjuvant treatment				<0.001
No	8479 (96)	7185 (97)	1294 (93)	
Yes*^1^*	341 (4)	250 (3)	91 (7)	
Adjuvant treatment				<0.001
No treatment	5874 (67)	5679 (76)	195 (14)	
RT	892 (10)	769 (10)	123 (9)	
Chemo	177 (2)	109 (2)	68 (5)	
RT+Chemo	1877 (21)	878 (12)	999 (72)	
ELN count*^2^*	16 (10, 23)	16 (9, 23)	17 (11, 25)	<0.001

All categorical variables were presented as numbers and proportions.

*^1^* including radiotherapy and/or chemotherapy.

*^2^* ELN count was presented as median with interquartile range.

*^3^* Pearson’s Chi-squared test; Wilcoxon rank sum test.

ADC, adenocarcinoma; SCC, squamous cell carcinoma; ASC, adenosquamous carcinoma; RT, radiotherapy; Chemo, chemotherapy; ELN, examined lymph node.

**Figure 2:**
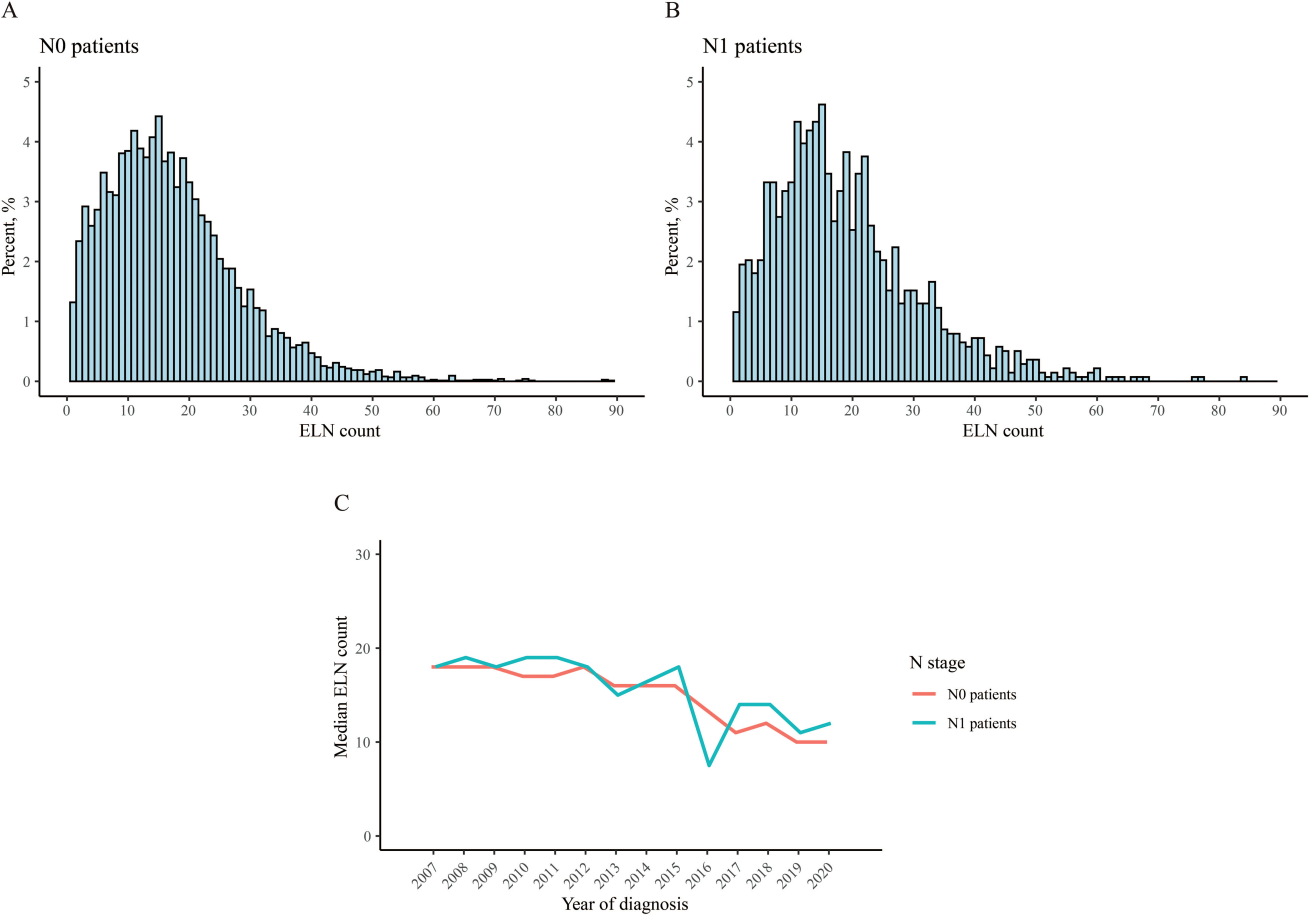
Distribution of the number of examined lymph node. **(A)** N0 patient, **(B)** N1 patient, **(C)** median count over years.

The distribution of ELN count in two groups were similar (**Figures 2A, B**), the median ELN counts were 16 and 17, respectively, and the IQR of N1 patients were bigger than N0 patients, with statistical significance (**Table 1**). The median ELN count decreased over years both in N0 patients and N1 patients (**Figure 2C**).

### The optimal cutoff value of ELN

**Figure 3:**
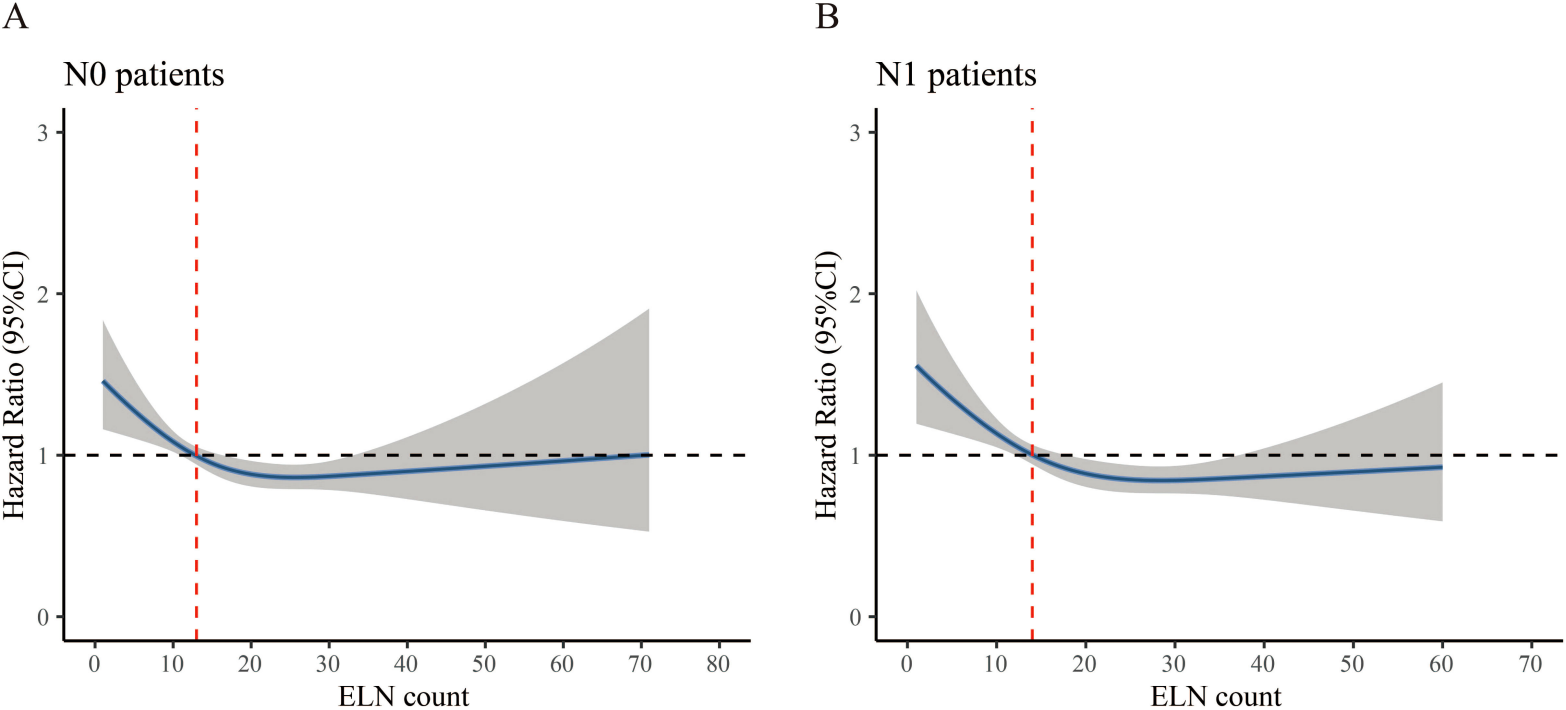
Restricted cubic spline curve associated with examined lymph node. **(A)** N0 patient, **(B)** N1 patient. Hazard ratio (HR) is indicated by blue solid line and 95% confidence interval by the shaded area. The red dashed line indicates the optimal cutoff value of examined lymph node (ELN). The horizontal dash line represents the point of no effect and is crucial for interpreting the clinical relevance of the ELN threshold. The point where the HR curve crosses or flattens around HR=1 indicates where additional lymph node retrieval may no longer confer further survival benefit.

To identify the prognostic profiles between ELN and CSS in resectable nonmetastatic cervical cancer patients, the RCS method was applied. For N0 patients, the RCS showed a nonlinear profile (nonlinearity, p= 0.026) predicting the survival, and the optimal cutoff value of ELN was 13 where the HR equal to or lower than 1 (**Figure 3A**). The curve was L-shape, and the risk of CSS decreased rapidly until an ELN count of 13 and afterwards. The results were adjusted for age, race, histology type, tumor grade, and tumor size. For N1 patients, the RCS also showed a nonlinear profile (nonlinearity, p= 0.024). The adjusted optimal cutoff value of ELN count was 14 for distinguishing those with high risk of death and those not (**Figure 3B**).

### Survival analysis according to optimal cutoff

**Figure 4:**
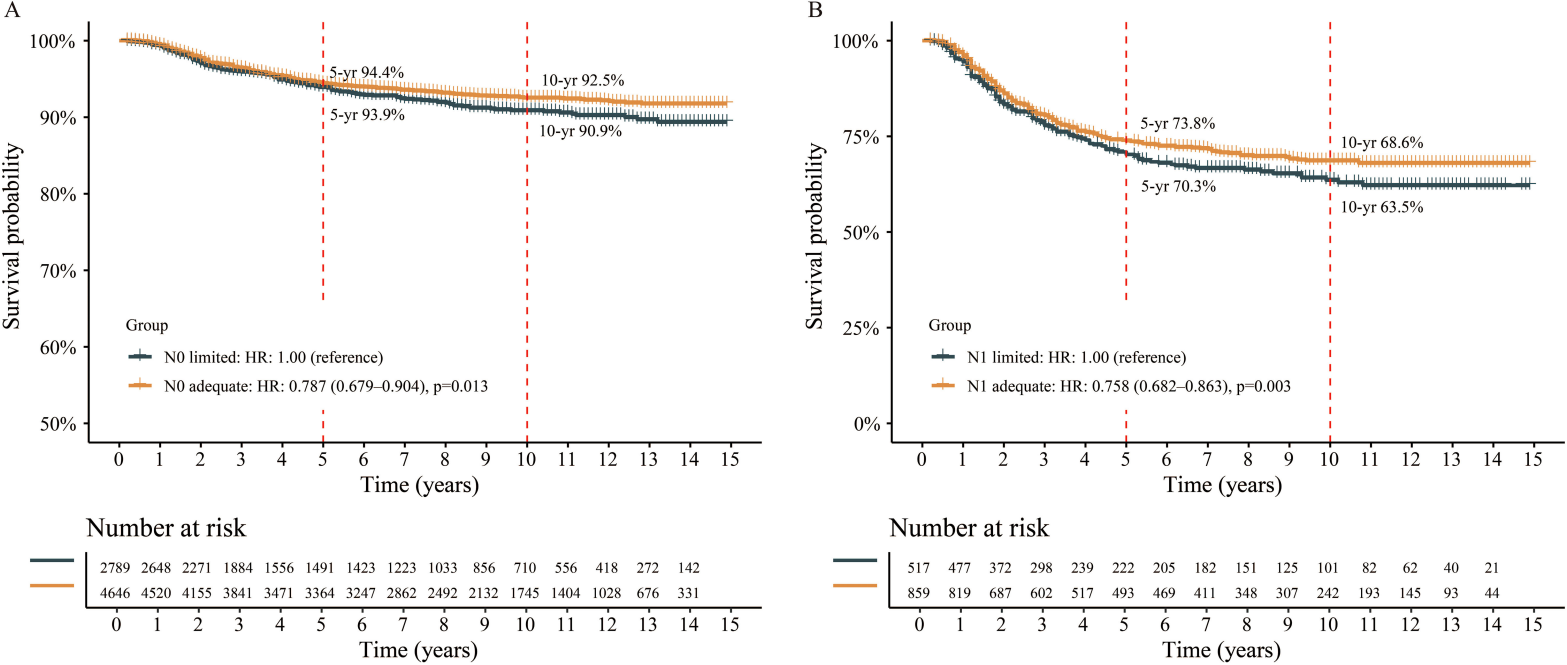
Survival analysis between different groups stratified by the optimal cutoff of examined lymph node. **(A)** N0 patient, **(B)** N1 patient.

To further explore the prognostic role of optimal cutoff of ELN count, all patients were divided into ELN count limited group or adequate group, and survival analyses were performed. In N0 stage, the median CSS of both groups were not reach. But those with adequate ELN count had better 5-yr (94.4% vs. 93.9%) and 10-yr (92.5% vs. 90.9%) CSS rates compared to those with limited ELN count (**Figure 4A**). The risk of death was 0.787 (95% CI 0.679-0.904, p=0.013) in N0 adequate patients when compared to N0 limited patients. The HRs remained lower when adjusted with neoadjuvant treatment, adjuvant treatment, and fully-adjusted (**Table 2**). In N1 stage, the same findings were obtained. The 5-yr and 10-yr survival rates were 73.8% and 68.6% for N1 adequate patients, and 70.3% and 63.5% for N1 limited patients (**Figure 4B**). The risk of death was 0.758 (N1 adequate vs. N1 limited, 95% CI 0.682-0.863, p=0.003) and remained lower after adjusted (**Table 2**). The details of multivariable Cox analysis were presented in **Supplementary Tables S1, S2**.

**Table 2:** Association of examined lymph node count with cancer-specific survival.

Group	Adjustment	HR (95% CI)	p-value
N0 patients	Non-adjusted	0.787 (0.679–0.904)	0.013
	Neoadjuvant-adjusted	0.817 (0.741–0.935)	0.025
	Adjuvant-adjusted	0.804 (0.684–0.913)	0.018
	Fully-adjusted	0.864 (0.714–0.952)	0.038
N1 patients	Non-adjusted	0.758 (0.682–0.863)	0.003
	Neoadjuvant-adjusted	0.814 (0.702–0.943)	0.021
	Adjuvant-adjusted	0.803 (0.686–0.936)	0.014
	Fully-adjusted	0.835 (0.747–0.956)	0.035

Patients with limited examined lymph node count as reference.

HR, hazard ratio; CI, confidence interval.

**Figure 5:**
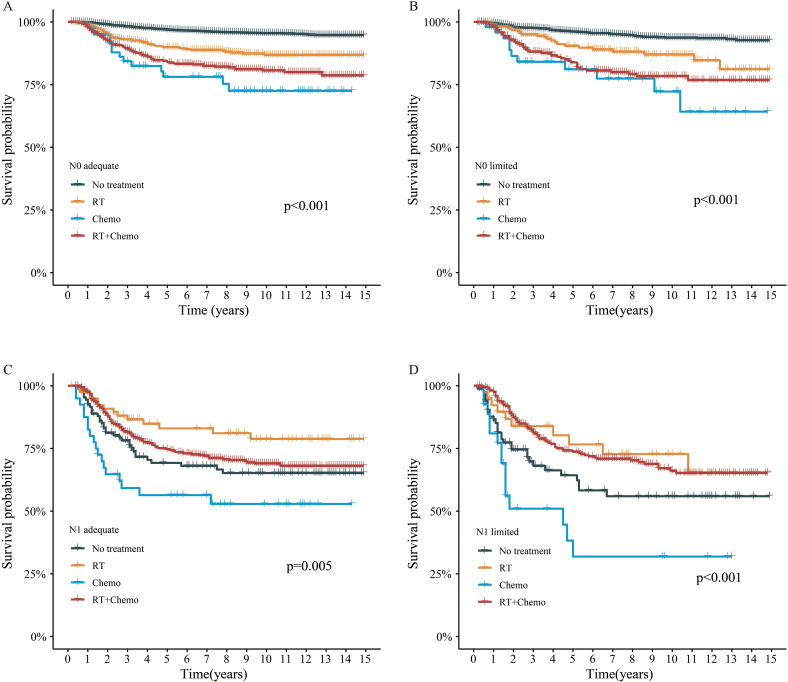
Survival analysis by adjuvant treatment. **(A)** N0 adequate patient, **(B)** N0 limited patient, **(C)** N1 adequate patient, **(D)** N1 limited patient.

Additionally, we explored the effect of different adjuvant treatments after surgery on CSS in each ELN subgroup. For both N0 adequate and N0 limited patients, no survival benefit was found in additional adjuvant treatment (**Figures 5A, B**). Even after adjusted, no survival benefit was obtained (**Table 3**). For N1 adequate patients, those with adjuvant RT obtained greatest survival benefit, followed by RT plus chemotherapy, no treatment, and chemotherapy only (**Figure 5C**, **Table 4**). For N1 limited patients, those with adjuvant RT or RT plus chemotherapy obtained better survival, followed by no treatment, and chemotherapy only (**Figure 5D**, **Table 4**). The details of multivariable Cox analysis were presented in **Supplementary Tables S3-S6**.

**Table 3:** Association of adjuvant treatment with cancer-specific survival in N0 patients.

Group	Adjustment	Subgroup*^1^*	HR (95% CI)	p-value
N0 adequate	Non-adjusted	RT	2.992 (2.179–4.109)	<0.001
		Chemo	6.654 (3.839–11.534)	<0.001
		RT+Chemo	4.812 (3.688–6.279)	<0.001
	Neoadjuvant-adjusted	RT	2.935 (2.137–4.032)	<0.001
		Chemo	6.264 (3.607–10.878)	<0.001
		RT+Chemo	4.678 (3.582–6.109)	<0.001
	Fully-adjusted	RT	1.738 (1.240–2.436)	0.001
		Chemo	3.128 (1.686–5.805)	<0.001
		RT+Chemo	2.352 (1.743–3.175)	<0.001
N0 limited	Non-adjusted	RT	2.324 (1.493–3.617)	<0.001
		Chemo	5.559 (2.974–10.39)	<0.001
		RT+Chemo	4.091 (2.911–5.75)	<0.001
	Neoadjuvant-adjusted	RT	2.441 (1.567–3.803)	<0.001
		Chemo	3.972 (2.076–7.598)	<0.001
		RT+Chemo	4.236 (3.012–5.958)	<0.001
	Fully-adjusted	RT	1.241 (0.777–1.981)	0.366
		Chemo	3.066 (1.597–5.885)	0.001
		RT+Chemo	1.903 (1.296–2.795)	0.001

*^1^* No adjuvant treatment as reference.

HR, hazard ratio; CI, confidence interval; RT, radiotherapy; Chemo, chemotherapy.

**Table 4:** Association of adjuvant treatment with cancer-specific survival in N1 patients.

Group	Adjustment	Subgroup*^1^*	HR (95% CI)	p-value
N1 adequate	Non-adjusted	No treatment	0.597 (0.337–1.056)	0.076
		RT	0.315 (0.157–0.633)	0.001
		RT+Chemo	0.494 (0.304–0.803)	0.004
	Neoadjuvant-adjusted	No treatment	0.603 (0.341–1.069)	0.083
		RT	0.327 (0.162–0.659)	0.002
		RT+Chemo	0.523 (0.319–0.857)	0.010
	Fully-adjusted	No treatment	0.584 (0.320–1.067)	0.080
		RT	0.381 (0.185–0.785)	0.009
		RT+Chemo	0.548 (0.328–0.916)	0.022
N1 limited	Non-adjusted	No treatment	0.494 (0.265–0.918)	0.026
		RT	0.286 (0.128–0.638)	0.002
		RT+Chemo	0.298 (0.173–0.516)	<0.001
	Neoadjuvant-adjusted	No treatment	0.484 (0.258–0.905)	0.023
		RT	0.288 (0.129–0.643)	0.002
		RT+Chemo	0.303 (0.175–0.525)	<0.001
	Fully-adjusted	No treatment	0.585 (0.305–1.120)	0.106
		RT	0.451 (0.194–1.047)	0.064
		RT+Chemo	0.396 (0.221–0.709)	0.002

*^1^* Adjuvant chemo as reference.

HR, hazard ratio; CI, confidence interval; RT, radiotherapy; Chemo, chemotherapy.

## Discussion

To the best of our knowledge, this is the first population-based study to explore the association between ELN count and long-term survival in resectable nonmetastatic cervical cancer patients. Our findings suggested that an ELN count of at least 13 in patients with clinical N0 stage, and 14 in those with clinical N1 stage, was associated with improved CSS outcomes.

The metastasis to LNM often indicated a poor prognosis. The FIGO 2018 staging and treatment guidelines recommend that patients with early-stage cervical cancer should undergo intraoperative pelvic lymph node dissection (LND), even if without LNM evidence evaluated by imaging examination (15). The rate of positive LNM was not high in early-stage cervical cancer underwent radical hysterectomy and lymphadenectomy, especially in those with no imaging evidence of positive lymph nodes (clinical N0 stage) (16, 17). In this study, we found that, among patients with cervical cancer and N0 stage, those with ELN counts greater than or equal to 13 had higher 5-yr and 10-yr CSS rates. These findings suggested that an adequate number of ELNs may play a positive role in achieving long-term survival benefits. Similarly, a study found that a minimum number of 20 ELNs was associated with progression-free survival in patients with stage IB1 to IIA cervical cancer (FIGO 2009) (5), especially in those with confirmed LNM after surgery. However, no association between ELN count and overall survival was found in the study. Pieterse et al. found that only in patients with positive nodes, a higher amount of ELN led to a better disease-free survival (6). Besides, Zhou et al. using ELN of 1-10, 11-20, 21-30, and >30 as cutoffs to classify patients into four groups, and found a positive association in SCC patients, but not ADC patients (7).

In this study, we found similar cutoff value of ELN count in N0 and N1 patients (13 and 14), which suggested lymphadenectomy was needed regardless of the imaging evidence of LNM, though the rate of LNM varied across different FIGO stages (17–19). The advantages of lymphadenectomy included reducing the risk of cancer cell dissemination, clarifying pathological staging for further adjuvant therapy, minimizing surgical extent and complications, as well as facilitating individualized treatment planning (20, 21). However, some researches indicated that excessive LND does not confer survival benefits to cervical cancer patients (22–24). Appropriate LND may have been integrated into clinical practice. Our study revealed a notable trend towards a gradual reduction in the median number of ELN over time. Specifically, from 2007 to 2020, the median ELN count decreased from 18 to 10 for N0 patients, and similarly, from 18 to 12 for N1 patients. However, our study also highlighted an L-shaped relationship between ELN and CSS, complicating the determination of an optimal maximum ELN count. Further exploration, potentially utilizing additional data or innovative statistical approaches, may be necessary to refine this issue.

Several established pathological features, such as tumor size, depth of stromal invasion, and lymphovascular space invasion (LVSI), are well-recognized prognostic factors in cervical cancer. For instance, Ayhan et al. identified tumor size, LVSI, and vaginal involvement as independent prognostic factors for disease-free and overall survival in FIGO stage IB patients without lymph node metastasis (25). Similarly, Huang et al. conducted a meta-analysis of over 25,000 patients and demonstrated that LVSI was significantly associated with decreased overall and disease-free survivals in stage IA–IIB cervical cancer, and remained an independent prognostic factor in multivariate analysis (26). Importantly, several studies suggested that the number of ELN and the presence of LVSI served as independent prognostic indicators, even when accounting for other pathological features. For example, Cao et al. found that tumor size >4 cm, LVSI positivity, and deep stromal invasion were independent risk factors for lymph node metastasis in early-stage cervical cancer (27). In a cohort of cervical cancer patients, Chen et al. found that a higher number of retrieved lymph nodes was significantly associated with improved progression-free survival, suggesting its role as a potential independent prognostic factor (28). Moreover, a recent retrospective study by Mereu et al. involving patients who received neoadjuvant chemotherapy (NACT) followed by radical surgery showed that tumor size, parametrial invasion, and LVSI were significant predictors of response, with the absence of LVSI associated with better lymph node status (29). Although NACT showed potential benefit in selected patients, it was not considered standard care, particularly for those with suspected nodal metastasis. Although our study was limited by the lack of detailed variables such as LVSI and invasion depth in the SEER dataset, we adjusted for available major clinical covariates. Our findings showed that adequate ELN counts was associated with improved survival, suggesting that the extent of lymph node examination might have prognostic value independent of other risk classifications, though further studies with comprehensive pathology data are warranted.

In recent years, the continual advancements and maturation of sentinel lymph node biopsy technology have emerged as a promising approach to acquiring precise and comprehensive information regarding LNM (30). This innovative method has the potential to mitigate the need for extensive lymph node resection procedures, including total pelvic and/or paraaortic lymphadenectomy, thereby benefiting patients by reducing unnecessary excessive surgical interventions.

Although an adequate count of ELN could bring survival advantages for N0 patients, further adjuvant treatments, including RT and chemotherapy, appeared unnecessary, even in those with limited ELN counts (**Figure 5B**). This result aligned with several prior studies, which likewise failed to demonstrate any survival benefit from adjuvant therapy in early-stage cervical cancer (31–33). These findings underscored that adjuvant treatment may be unnecessary in patients with no radiological evidence of LNM. For N1 patients with an adequate ELN count, those who received adjuvant RT alone experienced the most significant survival benefit, surpassing even those who underwent RT combined with chemotherapy. Consequently, it is advisable to administer adjuvant RT exclusively to this subset of patients. Among N1 patients with limited ELN, both adjuvant RT and adjuvant RT combined with chemotherapy exhibited comparable effects on CSS. Notably, for both N1 patients with adequate and limited ELN counts, adjuvant chemotherapy alone yielded dismal survival outcomes, inferior even to observation alone, indicating that adjuvant chemotherapy alone should not be recommended.

We acknowledged several limitations in this analysis. First, although we performed multivariate adjustments, the retrospective nature of the study introduced inherent selection bias that could not be fully eliminated. Second, the clinical significance of high ELN counts required further investigation. On one hand, the widening of the CIs beyond 30–40 ELNs in the RCS analysis (**Figure 3**) suggested a limited sample size in this range, indicating that conclusions regarding the survival impact of very high ELN counts should be interpreted with caution. On the other hand, excessive lymphadenectomy might have imposed additional surgical burden and disrupted lymphatic drainage, potentially leading to adverse effects on long-term survival. Third, key pathological variables such as LVSI and depth of stromal invasion were not available in the SEER database, which might have led to residual confounding in our adjusted analyses. Fourth, this study relied on imaging-based classification of lymph node status in the SEER database, which lacked detailed information on imaging modalities and diagnostic criteria. This may have introduced misclassification bias, particularly in borderline or micrometastatic cases. In addition, the absence of external validation further limited the generalizability of our findings. Future studies are warranted to assess the reproducibility of these results in independent datasets or single-center cohorts.

## Conclusion

In this population-based study, we found that patients with clinical N0 and N1 stages who had at least 13 and 14 ELN counts, respectively, tended to have better long-term survival outcomes. Moreover, the response to adjuvant therapy appeared to vary across ELN subgroups, indicating that the extent of lymph node evaluation may carry prognostic significance. Further prospective studies are needed to validate the association between ELN count and long-term survival.

## Data Availability

The raw data supporting the conclusions of this article will be made available by the authors, without undue reservation.
