# Supplementary material for: Association between examined lymph node count and survival in resectable cervical cancer: a retrospective analysis using SEER data
Source: Front Oncol. 2025 May 29;15:1553587. doi: 10.3389/fonc.2025.1553587 (PMC12158672; doi:10.3389/fonc.2025.1553587)
Supplement: Supplementary file 1 [file Table1.docx]

Table S1: Association of examined lymph node count with cancer-specific survival in N0 patients.

|  | **Variable** | **HR (95%CI)** | **p-value** |
| --- | --- | --- | --- |
| **Neoadjuvant-adjusted** | **N0 group** |  |  |
|  | N0 limited | Reference | – |
|  | N0 adequate | 0.817 (0.741–0.935) | 0.025 |
|  | **Neoadjuvant treatment** |  |  |
|  | No | Reference | – |
|  | Yes*^1^* | 0.897 (0.741–1.084) | 0.260 |
| **Adjuvant-adjusted** | **N0 group** |  |  |
|  | N0 limited | Reference | – |
|  | N0 adequate | 0.804 (0.684–0.913) | 0.018 |
|  | **Adjuvant treatment** |  |  |
|  | No treatment | Reference | – |
|  | RT | 2.728 (2.109–3.527) | <0.001 |
|  | Chemo | 6.187 (4.093–9.353) | <0.001 |
|  | RT+Chemo | 4.520 (3.666–5.574) | <0.001 |
| **Fully-adjusted** | **N0 group** |  |  |
|  | N0 limited | Reference | – |
|  | N0 adequate | 0.864 (0.714–0.952) | 0.038 |
|  | **Year of diagnosis** |  |  |
|  | 2007-2009 | Reference | – |
|  | 2010-2015 | 1.024 (0.834–1.256) | 0.823 |
|  | 2016-2020 | 0.912 (0.635–1.311) | 0.619 |
|  | **Age, years** |  |  |
|  | 15-45 | Reference | – |
|  | ≥45 | 1.263 (1.046–1.524) | 0.015 |
|  | **Race** |  |  |
|  | White | Reference | – |
|  | Black | 1.491 (1.137–1.955) | 0.004 |
|  | Other | 1.156 (0.887–1.505) | 0.284 |
|  | **Marital status** | |  |
|  | Married | Reference | – |
|  | Ever | 1.170 (0.913–1.497) | 0.214 |
|  | Single | 1.214 (0.978–1.508) | 0.079 |
|  | Other | 0.829 (0.498–1.382) | 0.473 |
|  | **Histology type** |  |  |
|  | ADC | Reference | – |
|  | SCC | 1.171 (0.929–1.476) | 0.181 |
|  | ASC | 1.851 (1.296–2.644) | 0.001 |
|  | Other | 2.399 (1.549–3.716) | <0.001 |
|  | **Tumor grade** |  |  |
|  | Grade I | Reference | – |
|  | Grade II | 1.384 (0.967–1.983) | 0.076 |
|  | Grade III | 1.801 (1.244–2.607) | 0.002 |
|  | Grade IV | 2.056 (1.127–3.749) | 0.019 |
|  | Unknown | 0.871 (0.553–1.373) | 0.553 |
|  | **Tumor size, mm** |  |  |
|  | ≤ 20 | Reference | – |
|  | 21–40 | 2.288 (1.772–2.954) | <0.001 |
|  | > 40 | 3.391 (2.564–4.485) | <0.001 |
|  | Unknown | 1.177 (0.827–1.674) | 0.366 |
|  | **Neoadjuvant treatment** |  |  |
|  | No | Reference | – |
|  | Yes*^1^* | 0.860 (0.709–1.043) | 0.126 |
|  | **Adjuvant treatment** |  |  |
|  | No treatment | Reference | – |
|  | RT | 1.559 (1.187–2.046) | 0.001 |
|  | Chemo | 2.972 (1.907–4.632) | <0.001 |
|  | RT+Chemo | 2.197 (1.738–2.777) | <0.001 |
| *^1^* including radiotherapy and/or chemotherapy | | | |
| HR, hazard ratio; CI, confidence interval; ADC, adenocarcinoma; SCC, squamous cell carcinoma; ASC, adenosquamous carcinoma; RT, radiotherapy; Chemo, chemotherapy. | | | |

Table S2: Association of examined lymph node count with cancer-specific survival in N1 patients.

|  | **Variable** | **HR (95%CI)** | **p-value** |
| --- | --- | --- | --- |
| **Neoadjuvant**-**adjusted** | **N1 group** |  |  |
|  | N1 limited | Reference | – |
|  | N1 adequate | 0.814 (0.702–0.943) | 0.021 |
|  | **Neoadjuvant treatment** |  |  |
|  | No | Reference | – |
|  | Yes*^1^* | 1.251 (0.692–1.847) | 0.127 |
| **Adjuvant**-**adjusted** | **N1 group** |  |  |
|  | N1 limited | Reference | – |
|  | N1 adequate | 0.803 (0.686–0.936) | 0.014 |
|  | **Adjuvant treatment** |  |  |
|  | No treatment | Reference | – |
|  | RT | 0.541 (0.339–0.866) | 0.010 |
|  | Chemo | 1.801 (1.183–2.741) | 0.006 |
|  | RT+Chemo | 0.726 (0.553–0.955) | 0.022 |
| **Fully-adjusted** | **N1 group** |  |  |
|  | N1 limited | Reference | – |
|  | N1 adequate | 0.835 (0.747–0.956) | 0.035 |
|  | **Year of diagnosis** |  |  |
|  | 2007-2009 | Reference | – |
|  | 2010-2015 | 1.107 (0.880–1.392) | 0.386 |
|  | 2016-2020 | 0.827 (0.559–1.224) | 0.343 |
|  | **Age, years** |  |  |
|  | 15-45 | Reference | – |
|  | ≥45 | 1.125 (0.908–1.393) | 0.281 |
|  | **Race** |  |  |
|  | White | Reference | – |
|  | Black | 1.209 (0.841–1.738) | 0.306 |
|  | Other | 0.842 (0.621–1.143) | 0.271 |
|  | **Marital status** | |  |
|  | Married | Reference | – |
|  | Ever | 1.350 (1.036–1.758) | 0.026 |
|  | Single | 0.917 (0.710–1.184) | 0.506 |
|  | Other | 1.438 (0.861–2.401) | 0.165 |
|  | **Histology type** |  |  |
|  | ADC | Reference | – |
|  | SCC | 0.606 (0.473–0.778) | <0.001 |
|  | ASC | 0.937 (0.635–1.384) | 0.745 |
|  | Other | 1.511 (0.992–2.301) | 0.055 |
|  | **Tumor grade** |  |  |
|  | Grade I | Reference | – |
|  | Grade II | 0.886 (0.537–1.464) | 0.638 |
|  | Grade III | 1.315 (0.796–2.173) | 0.286 |
|  | Grade IV | 1.516 (0.778–2.952) | 0.222 |
|  | Unknown | 1.260 (0.707–2.247) | 0.433 |
|  | **Tumor size, mm** |  |  |
|  | ≤ 20 | Reference | – |
|  | 21-40 | 1.974 (1.311–2.973) | 0.001 |
|  | > 40 | 3.019 (2.012–4.530) | <0.001 |
|  | Unknown | 1.837 (1.104–3.058) | 0.019 |
|  | **Neoadjuvant treatment** |  |  |
|  | No | Reference | – |
|  | Yes*^1^* | 1.217 (0.833–1.781) | 0.310 |
|  | **Adjuvant treatment** |  |  |
|  | No treatment | Reference | – |
|  | RT | 0.654 (0.407–1.053) | 0.080 |
|  | Chemo | 1.540 (1.001–2.368) | 0.049 |
|  | RT+Chemo | 0.790 (0.592–1.055) | 0.111 |
| *^1^* including radiotherapy and/or chemotherapy | | | |
| HR, hazard ratio; CI, confidence interval; ADC, adenocarcinoma; SCC, squamous cell carcinoma; ASC, adenosquamous carcinoma; RT, radiotherapy; Chemo, chemotherapy. | | | |

Table S3: Association of adjuvant treatment with cancer-specific survival in N0 adequate patients.

|  | **Variable** | **HR (95%CI)** | **p-value** |
| --- | --- | --- | --- |
| **Neoadjuvant-adjusted** | **Adjuvant treatment** |  |  |
|  | No treatment | Reference | – |
|  | RT | 2.935 (2.137–4.032) | <0.001 |
|  | Chemo | 6.264 (3.607–10.878) | <0.001 |
|  | RT+Chemo | 4.678 (3.582–6.109) | <0.001 |
|  | **Neoadjuvant treatment** |  |  |
|  | No | Reference | – |
|  | Yes*^1^* | 0.893 (0.721–1.105) | 0.296 |
| **Fully-adjusted** | **Adjuvant treatment** |  |  |
|  | No treatment | Reference | – |
|  | RT | 1.738 (1.240–2.436) | 0.001 |
|  | Chemo | 3.128 (1.686–5.805) | <0.001 |
|  | RT+Chemo | 2.352 (1.743–3.175) | <0.001 |
|  | **Year of diagnosis** |  |  |
|  | 2007-2009 | Reference | – |
|  | 2010-2015 | 0.956 (0.742–1.233) | 0.730 |
|  | 2016-2020 | 1.065 (0.640–1.772) | 0.808 |
|  | **Age, years** |  |  |
|  | 15-45 | Reference | – |
|  | ≥45 | 1.035 (0.815–1.313) | 0.779 |
|  | **Race** |  |  |
|  | White | Reference | – |
|  | Black | 1.300 (0.888–1.903) | 0.177 |
|  | Other | 0.982 (0.693–1.391) | 0.918 |
|  | **Marital status** | |  |
|  | Married | Reference | – |
|  | Ever | 1.167 (0.849–1.604) | 0.342 |
|  | Single | 1.187 (0.902–1.561) | 0.222 |
|  | Other | 1.000 (0.552–1.812) | 0.999 |
|  | **Histology type** |  |  |
|  | ADC | Reference | – |
|  | SCC | 1.144 (0.852–1.537) | 0.370 |
|  | ASC | 1.519 (0.941–2.452) | 0.087 |
|  | Other | 2.696 (1.532–4.742) | 0.001 |
|  | **Tumor grade** |  |  |
|  | Grade I | Reference | – |
|  | Grade II | 1.429 (0.913–2.237) | 0.118 |
|  | Grade III | 1.804 (1.132–2.876) | 0.013 |
|  | Grade IV | 2.466 (1.198–5.074) | 0.014 |
|  | Unknown | 0.606 (0.315–1.166) | 0.134 |
|  | **Tumor size, mm** |  |  |
|  | ≤ 20 | Reference | – |
|  | 21-40 | 1.973 (1.442–2.700) | <0.001 |
|  | > 40 | 3.043 (2.154–4.299) | <0.001 |
|  | Unknown | 1.168 (0.746–1.830) | 0.496 |
|  | **Neoadjuvant treatment** |  |  |
|  | No | Reference | – |
|  | Yes*^1^* | 0.975 (0.741–1.448) | 0.389 |
| *^1^* including radiotherapy and/or chemotherapy | | | |
| HR, hazard ratio; CI, confidence interval; ADC, adenocarcinoma; SCC, squamous cell carcinoma; ASC, adenosquamous carcinoma; RT, radiotherapy; Chemo, chemotherapy. | | | |

Table S4: Association of adjuvant treatment with cancer-specific survival in N0 limited patients.

|  | **Variable** | **HR (95%CI)** | **p-value** |
| --- | --- | --- | --- |
| **Neoadjuvant-adjusted** | **Adjuvant treatment** |  |  |
|  | No treatment | Reference | – |
|  | RT | 2.441 (1.567–3.803) | <0.001 |
|  | Chemo | 3.972 (2.076–7.598) | <0.001 |
|  | RT+Chemo | 4.236 (3.012–5.958) | <0.001 |
|  | **Neoadjuvant treatment** |  |  |
|  | No | Reference | – |
|  | Yes*^1^* | 1.210 (0.776–1.535) | 0.271 |
| **Fully-adjusted** | **Adjuvant treatment** |  |  |
|  | No treatment | Reference | – |
|  | RT | 1.241 (0.777–1.981) | 0.366 |
|  | Chemo | 3.066 (1.597–5.885) | 0.001 |
|  | RT+Chemo | 1.903 (1.296–2.795) | 0.001 |
|  | **Year of diagnosis** |  |  |
|  | 2007-2009 | Reference | – |
|  | 2010-2015 | 1.148 (0.807–1.632) | 0.444 |
|  | 2016-2020 | 0.859 (0.503–1.466) | 0.576 |
|  | **Age, years** |  |  |
|  | 15-45 | Reference | – |
|  | ≥45 | 1.717 (1.251–2.357) | 0.001 |
|  | **Race** |  |  |
|  | White | Reference | – |
|  | Black | 1.804 (1.209–2.693) | 0.004 |
|  | Other | 1.585 (1.046–2.402) | 0.030 |
|  | **Marital status** | |  |
|  | Married | Reference | – |
|  | Ever | 1.189 (0.796–1.776) | 0.399 |
|  | Single | 1.374 (0.963–1.960) | 0.080 |
|  | Other | 0.622 (0.226–1.71) | 0.357 |
|  | **Histology type** |  |  |
|  | ADC | Reference | – |
|  | SCC | 1.186 (0.813–1.729) | 0.377 |
|  | ASC | 2.423 (1.408–4.169) | 0.001 |
|  | Other | 1.912 (0.913–4.006) | 0.086 |
|  | **Tumor grade** |  |  |
|  | Grade I | Reference | – |
|  | Grade II | 1.374 (0.750–2.520) | 0.304 |
|  | Grade III | 1.891 (1.025–3.488) | 0.042 |
|  | Grade IV | 1.951 (0.629–6.051) | 0.247 |
|  | Unknown | 1.202 (0.607–2.380) | 0.597 |
|  | **Tumor size, mm** |  |  |
|  | ≤ 20 | Reference | – |
|  | 21-40 | 2.975 (1.903–4.650) | <0.001 |
|  | > 40 | 4.504 (2.770–7.324) | <0.001 |
|  | Unknown | 1.265 (0.709–2.257) | 0.427 |
|  | **Neoadjuvant treatment** |  |  |
|  | No | Reference | – |
|  | Yes*^1^* | 1.447 (0.908–2.304) | 0.120 |
| *^1^* including radiotherapy and/or chemotherapy | | | |
| HR, hazard ratio; CI, confidence interval; ADC, adenocarcinoma; SCC, squamous cell carcinoma; ASC, adenosquamous carcinoma; RT, radiotherapy; Chemo, chemotherapy. | | | |

Table S5: Association of adjuvant treatment with cancer-specific survival in N1 adequate patients.

|  | **Variable** | **HR (95%CI)** | **p-value** |
| --- | --- | --- | --- |
| **Neoadjuvant-adjusted** | **Adjuvant treatment** |  |  |
|  | Chemo | Reference | – |
|  | No treatment | 0.603 (0.341–1.069) | 0.083 |
|  | RT | 0.327 (0.162–0.659) | 0.002 |
|  | RT+Chemo | 0.523 (0.319–0.857) | 0.010 |
|  | **Neoadjuvant treatment** |  |  |
|  | No | Reference | – |
|  | Yes*^1^* | 1.455 (0.875–2.421) | 0.149 |
| **Fully-adjusted** | **Adjuvant treatment** |  |  |
|  | Chemo | Reference | – |
|  | No treatment | 0.584 (0.320–1.067) | 0.080 |
|  | RT | 0.381 (0.185–0.785) | 0.009 |
|  | RT+Chemo | 0.548 (0.328–0.916) | 0.022 |
|  | **Year of diagnosis** |  |  |
|  | 2007-2009 | Reference | – |
|  | 2010-2015 | 0.986 (0.738–1.318) | 0.925 |
|  | 2016-2020 | 0.798 (0.456–1.397) | 0.430 |
|  | **Age, years** |  |  |
|  | 15-45 | Reference | – |
|  | ≥45 | 1.155 (0.879–1.518) | 0.302 |
|  | **Race** |  |  |
|  | White | Reference | – |
|  | Black | 1.998 (1.236–3.231) | 0.005 |
|  | Other | 0.903 (0.610–1.337) | 0.610 |
|  | **Marital status** | |  |
|  | Married | Reference | – |
|  | Ever | 1.265 (0.893–1.791) | 0.186 |
|  | Single | 0.884 (0.640–1.221) | 0.455 |
|  | Other | 1.104 (0.528–2.309) | 0.792 |
|  | **Histology type** |  |  |
|  | ADC | Reference | – |
|  | SCC | 0.597 (0.434–0.822) | 0.002 |
|  | ASC | 0.967 (0.605–1.546) | 0.888 |
|  | Other | 1.550 (0.878–2.737) | 0.131 |
|  | **Tumor grade** |  |  |
|  | Grade I | Reference | – |
|  | Grade II | 1.061 (0.519–2.166) | 0.871 |
|  | Grade III | 1.702 (0.834–3.471) | 0.144 |
|  | Grade IV | 1.937 (0.772–4.862) | 0.159 |
|  | Unknown | 1.501 (0.646–3.487) | 0.345 |
|  | **Tumor size, mm** |  |  |
|  | ≤ 20 | Reference | – |
|  | 21-40 | 2.411 (1.364–4.262) | 0.002 |
|  | > 40 | 3.852 (2.185–6.792) | <0.001 |
|  | Unknown | 1.748 (0.859–3.557) | 0.123 |
|  | **Neoadjuvant treatment** |  |  |
|  | No | Reference | – |
|  | Yes*^1^* | 1.387 (0.823–2.338) | 0.219 |
| *^1^* including radiotherapy and/or chemotherapy | | | |
| HR, hazard ratio; CI, confidence interval; ADC, adenocarcinoma; SCC, squamous cell carcinoma; ASC, adenosquamous carcinoma; RT, radiotherapy; Chemo, chemotherapy. | | | |

Table S6: Association of adjuvant treatment with cancer-specific survival in N1 limited patients.

|  | **Variable** | **HR (95%CI)** | **p-value** |
| --- | --- | --- | --- |
| **Neoadjuvant-adjusted** | **Adjuvant treatment** |  |  |
|  | Chemo | Reference | – |
|  | No treatment | 0.484 (0.258–0.905) | 0.023 |
|  | RT | 0.288 (0.129–0.643) | 0.002 |
|  | RT+Chemo | 0.303 (0.175–0.525) | <0.001 |
|  | **Neoadjuvant treatment** |  |  |
|  | No | Reference | – |
|  | Yes*^1^* | 1.147 (0.661–1.990) | 0.626 |
| **Fully-adjusted** | **Adjuvant treatment** |  |  |
|  | Chemo | Reference | – |
|  | No treatment | 0.585 (0.305–1.120) | 0.106 |
|  | RT | 0.451 (0.194–1.047) | 0.064 |
|  | RT+Chemo | 0.396 (0.221–0.709) | 0.002 |
|  | **Year of diagnosis** |  |  |
|  | 2007-2009 | Reference | – |
|  | 2010-2015 | 1.333 (0.885–2.006) | 0.169 |
|  | 2016-2020 | 0.868 (0.478–1.577) | 0.642 |
|  | **Age, years** |  |  |
|  | 15-45 | Reference | – |
|  | ≥45 | 1.120 (0.787–1.592) | 0.529 |
|  | **Race** |  |  |
|  | White | Reference | – |
|  | Black | 0.764 (0.432–1.353) | 0.356 |
|  | Other | 0.729 (0.440–1.208) | 0.220 |
|  | **Marital status** | |  |
|  | Married | Reference | – |
|  | Ever | 1.441 (0.941–2.207) | 0.093 |
|  | Single | 0.916 (0.593–1.416) | 0.693 |
|  | Other | 1.752 (0.839–3.658) | 0.135 |
|  | **Histology type** |  |  |
|  | ADC | Reference | – |
|  | SCC | 0.682 (0.453–1.026) | 0.066 |
|  | ASC | 0.849 (0.399–1.807) | 0.672 |
|  | Other | 1.638 (0.847–3.166) | 0.143 |
|  | **Tumor grade** |  |  |
|  | Grade I | Reference | – |
|  | Grade II | 0.689 (0.329–1.444) | 0.324 |
|  | Grade III | 0.874 (0.413–1.851) | 0.725 |
|  | Grade IV | 1.052 (0.376–2.944) | 0.923 |
|  | Unknown | 1.037 (0.458–2.347) | 0.931 |
|  | **Tumor size, mm** |  |  |
|  | ≤ 20 | Reference | – |
|  | 21-40 | 1.463 (0.793–2.700) | 0.223 |
|  | > 40 | 2.212 (1.208–4.051) | 0.010 |
|  | Unknown | 2.110 (0.986–4.519) | 0.055 |
|  | **Neoadjuvant treatment** |  |  |
|  | No | Reference | – |
|  | Yes*^1^* | 1.120 (0.628–1.997) | 0.702 |
| *^1^* including radiotherapy and/or chemotherapy | | | |
| HR, hazard ratio; CI, confidence interval; ADC, adenocarcinoma; SCC, squamous cell carcinoma; ASC, adenosquamous carcinoma; RT, radiotherapy; Chemo, chemotherapy. | | | |
